# Early remission in multiple sclerosis is linked to altered coherence of the Cerebellar Network

**DOI:** 10.1186/s12967-022-03576-4

**Published:** 2022-10-27

**Authors:** Marlene Tahedl, Seth M. Levine, Robert Weissert, Zacharias Kohl, De-Hyung Lee, Ralf A. Linker, Jens V. Schwarzbach

**Affiliations:** 1 Department of Psychiatry and Psychotherapy, University of Regensburg, 93053 Regensburg, Germany; 2 Institute for Psychology, University of Regensburg, 93053 Regensburg, Germany; 3 Department of Psychology, LMU Munich, 80802 Munich, Germany; 4 NeuroImaging Core Unit Munich (NICUM), University Hospital LMU, 80336 Munich, Germany; 5 Department of Neurology, University of Regensburg, 93053 Regensburg, Germany

**Keywords:** Cerebellar Network, Functional connectivity, Independent component analysis, Multiple sclerosis, Remission

## Abstract

**Background:**

The development of permanent disability in multiple sclerosis (MS) is highly variable among patients, and the exact mechanisms that contribute to this disability remain unknown.

**Methods:**

Following the idea that the brain has intrinsic network organization, we investigated changes of functional networks in MS patients to identify possible links between network reorganization and remission from clinical episodes in MS. Eighteen relapsing–remitting MS patients (RRMS) in their first clinical manifestation underwent resting-state functional MRI and again during remission. We used ten template networks, identified from independent component analysis, to compare changes in network coherence for each patient compared to those of 44 healthy controls from the Human Connectome Project test–retest dataset (two-sample t-﻿test of pre-post differences). Combining a binomial test with Monte Carlo procedures, we tested four models of how functional coherence might change between the first clinical episode and remission: a network can change its coherence (a) with itself (“one-with-self”), (b) with another network (“one-with-other”), or (c) with a set of other networks (“one-with-many”), or (d) multiple networks can change their coherence with respect to one common network (“many-with-one”).

**Results:**

We found evidence supporting two of these hypotheses: coherence decreased between the Executive Control Network and several other networks (“one-with-many” hypothesis), and a set of networks altered their coherence with the Cerebellar Network (“many-with-one” hypothesis).

**Conclusion:**

Given the unexpected commonality of the Cerebellar Network’s altered coherence with other networks (a finding present in more than 70% of the patients, despite their clinical heterogeneity), we conclude that remission in MS may result from learning processes mediated by the Cerebellar Network.

**Supplementary Information:**

The online version contains supplementary material available at 10.1186/s12967-022-03576-4.

## Introduction

In multiple sclerosis (MS), the most commonly observed disease course is characterized by an alternating pattern of new or enhanced neurological symptoms (“relapses”) and phases of partial or complete recovery (“remission”). These clinical episodes often co-occur with an acute inflammatory insult, causing demyelination and neurodegeneration to the central nervous system (CNS). Such sudden injury is believed to be the source of the symptoms, as demyelination hampers signal transmission [1]. Consequently, given the network nature of the brain, which relies on complex systemwide communication to carry out tasks [2], it is reasonable to assume that any interruption of signal transmission results in suboptimal task performance, which can present as neurological symptoms [3]. Although during remission the inflammation has subsided, sclerotic, demyelinated plaques remain [4]; nevertheless, patients are often completely symptom-free, especially following early relapses [5]. Therefore, it would appear as though the brain employs mechanisms beyond lesion repair in order to regain and maintain function following lesion occurrence.

Classic work has shown that the brain is able to flexibly reorganize (generally referred to as “neuroplasticity” [6, 7]), even in elderly people [8]. Studies using functional magnetic resonance imaging (fMRI) have demonstrated that the neural correlates underlying specific tasks differ between MS patients and healthy controls (HCs), thereby suggesting neuroplastic reorganization. For example, Pantano and colleagues [9] investigated the neural correlates of hand movements in MS patients with an ipsi- as well as a contralateral hemiparesis. This study showed that, even in the absence of any clinical manifestation, the same task implicates different cortical areas in MS patients compared to HCs. Additionally, functional reorganization in MS patients has been observed at clinical recovery after pseudotumoral lesions [10]. Related observations have been reported by other similar studies [11–13].

One drawback of such task-based fMRI is that its findings can generally only be interpreted within the context of specific tasks that probe specific functions. However, specific tasks that uncover large-scale changes in network organization between a heterogeneous group of MS-patients and a group of HCs are not known or may not exist. Therefore, examining network properties speaks in favor of employing resting-state fMRI (rs-fMRI), which allows the analysis of network-level activity in the brain in an unconstrained manner [14]. Many investigations have looked into the reorganization of resting-state networks in MS patients, indeed demonstrating some general, fundamental differences in this organization compared to HCs (e.g. [15, 16], for a review see [17]). However, such studies do not address the mechanisms that underlie successful or failed remission.

**Fig. 1:**
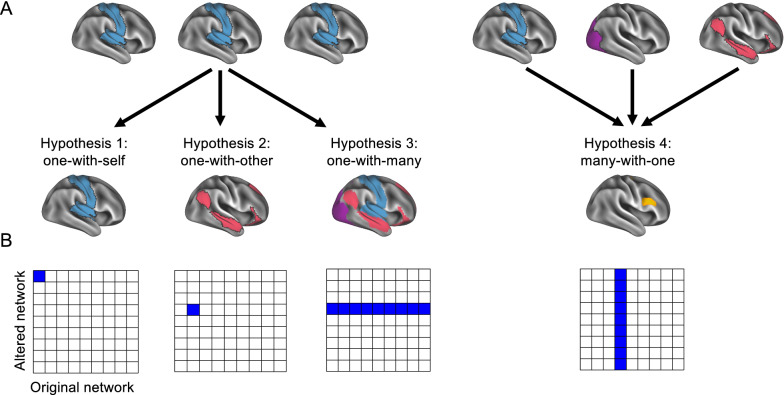
Hypotheses. Conceptual representation of the hypotheses depicted as **A** brain maps and **B** adjacency matrices. We hypothesize that functional network coherence changes between relapse and remission can occur as changes from an original network to itself (one-with-self), to another network (one-with-other), or to a set of networks (one-with-many), or that regardless of the original network, these networks change with respect to a common network (many-with-one)

In this study, we recruited MS patients suffering from their first clinical manifestation. Our approach sought to investigate the changes in functional coherence (a type of functional connectivity, [14]) of brain networks between the first episode, during which patients exhibited neurological symptoms, and remission (when they were symptom-free, on average four weeks after the first scan). With this experimental design, we investigated the following questions: although MS is a highly heterogeneous disease, is there nevertheless a common brain network that changes in all MS patients between a clinical episode and remission? If so, does this network change with respect to itself (1) a one-with-self change), with respect to a specific other network (2) a one-with-other change), or with respect to a variety of other networks (3) a one-with-many change)? Furthermore, is there a particular region of the brain (or set of regions) to which patients’ networks systematically change their coherence (4) a many-with-one change)? These hypotheses (Fig. 1) follow the idea of a putative general mechanism driving the recovery toward remission [18] but implicate different physiological levels with respect to the function of remission in MS.

## Methods

### Participants

#### Patients

For this study, we recruited 18 relapsing–remitting MS patients (14 females, 4 males, age (mean + /– standard deviation) = 28.33 + /– 9.36 years) in their initial clinical episode (diagnosed according to the 2017 revisions of the McDonald criteria [19]: dissemination in space was demonstrated by MRI, dissemination in time by presence of oligoclonal bands). Each patient was scanned twice: once during the first episode [i.e., with ongoing presentation of symptoms (EDSS ≥ 1 in all cases)] and the second time during remission (when we expected a decrease in EDSS scores, on average four weeks after the first scan). The patients showed different clinical symptoms (Table 1). For six patients, no structured EDSS assessment was available in remission. We calculated the median EDSS scores during the first clinical episode and remission and used a Wilcoxon signed-rank test to test the null hypothesis that median ranks from both time points are equal. We calculated T2 lesion load for all but one patient (for whom no T2 FLAIR image was available from the first session due to excessive motion) in both sessions using the Lesion Prediction Algorithm of the Lesion Segmentation Toolbox [20] and assessed changes of lesion load between the two scans with a paired t-test. All patients provided written informed consent to participate in this study. All study procedures followed safety guidelines for MRI research at the University of Regensburg, complied with the 1964 Helsinki Declaration and its later amendments, and were approved by the ethics committee of the University of Regensburg.

**Table 1:** Demographic and clinical data of the patients

Patient ID	Age / Sex	T2 LL (relapse)	T2 LL (remission)	EDSS score (relapse)	EDSS score (remission)	Neurological symptoms
MS01	28 / f	1.72 ml	1.30 ml	3.0	1.0	Internuclear ophthalmoplegia
MS02	23 / f	3.93 ml	6.06 ml	2.0	n.a. **	Facial nerve paralysis
MS03	54 / f	19.13 ml	21.61 ml	3.0	1.5	Paresis left leg, restricted walking range, limb ataxia
MS04	21 / f	0.36 ml	0.36 ml	2.0	1.5	Optic neuritis
MS05	23 / f	14.41 ml	13.42 ml	2.0	1.0	Optic neuritis, facial nerve paralysis, mild fatigue, sensory disturbances
MS06	27 / f	n.a.*	1.53 ml	3.0	n.a. **	Optic neuritis, sensory disturbances
MS07	29 / m	0.85 ml	0.69 ml	2.5	1.0	Gait ataxia, sensory disturbances
MS08	22 / f	0.02 ml	0.14 ml	1.0	1.0	Optic neuritis
MS09	22 / f	1.41 ml	0.41 ml	2.0	0.0	Optic neuritis
MS10	21 / f	1.93 ml	1.62 ml	3.0	2.0	Optic neuritis
MS11	24 / f	8.97 ml	3.30 ml	3.0	n.a. **	Tetraparesis, gait ataxia
MS12	35 / m	0.44 ml	0.46 ml	2.5	n.a. **	Cerebellar ataxia
MS13	33 / f	4.58 ml	4.81 ml	2.0	n.a. **	Slight ataxia, paresis of the left leg
MS14	18 / m	1.28 ml	0.94 ml	1.0	1.0	Vertigo, gait ataxia
MS15	23 / f	0.03 ml	0.02 ml	2.0	1.0	Optic neuritis
MS16	35 / m	0.45 ml	1.41 ml	2.5	3.0	Sensory disturbances, fatigue
MS17	26 / f	1.67 ml	1.62 ml	2.0	n.a. **	Optic neuritis
MS18	46 / f	0.19 ml	0.16 ml	4.0	1.0	Optic neuritis

^*^No T2 FLAIR image was available at relapse due to excessive motion of the patient during that sequence

^**^No structured EDSS assessment was available in remission

*EDSS* expanded disability status scale, *LL* lesion load, *n.a.*  not available

#### Healthy control participants

We used data from the Human Connectome Project 1200 Subjects Data Release (www.humanconnectome.org) as a control dataset [21, 22]. Specifically, we made use of the minimally-preprocessed rs-fMRI data, which includes complete test–retest data from 44 participants (30 females, age range = 22–35 years). Participants were scanned on two separate days, separated by an interscan interval between 3 and 11 months. All study procedures of the HCP protocol were approved by the Institutional Review Board at Washington University in St. Louis.

#### Neuroimaging data acquisition

Data acquisition of the 18 MS patients was carried out on a 3 Tesla Prisma scanner (Siemens, Erlangen, Germany). Resting-state functional images were acquired with a T2*-weighted EPI sequence (instruction to participants: eyes closed, repetition time (TR) = 2000 ms, echo time (TE) = 30.0 ms, flip angle (FA) = 70°, field of view (FOV) = 192 × 192 mm^2^, matrix size = 64 × 64, voxel resolution (VR) = 3 × 3 × 3.6 mm^3^, 33 axial slices (acquired ascending-interleaved), phase encoding direction = anterior–posterior, echo spacing = 0.58 ms, pixel bandwidth (BW) = 2605 Hz/Px, scan duration = 22 min 6 s, 660 volumes per run; three dummy volumes were acquired at the beginning of the scan to account for T1-saturation). For co-registration of the functional data to high-resolution native anatomical space, we acquired a T1-weighted scan using a 3D Turboflash MPRAGE sequence (TR = 1910 ms, TE = 3.67 ms, inversion time (TI) = 1040 ms, 180 sagittal slices, FA = 9°, FOV = 250 × 250 mm^2^, matrix size = 256 × 256, VR = 0.98 × 0.98 × 1 mm^3^, BW = 180 Hz/Px, scan duration = 4 min 25 s). Additionally, to quantify lesion load, we acquired a T2-weighted 2D Fluid Attenuated Inversion Recovery (FLAIR) image (TR = 9000 ms, TE = 108 ms, TI = 2500 ms, 27 axial slices, FA = 150°, FOV = 186 × 230  mm^2^, matrix size = 260 × 320, VR = 0.72 × 0.72 × 5.5 mm^3^, BW = 290 Hz/Px, scan duration = 2 min 14 s) and calculated the lesion load using an automatic lesion-segmentation method [20].

The HCP data was acquired on a 3 Tesla Connectome Skyra Scanner (instruction to participants: eyes closed, TR = 720 ms, TE = 33.1 ms, FA = 52°, FOV = 208 × 180mm^2^, matrix size = 104 × 90, voxel resolution = 2 × 2 × 2 mm^3^, 72 slices, phase encoding direction = right–left, multiband factor = 8, echo spacing = 0.58 ms, BW = 2290 Hz/Px, scan duration = 14 min 33 s, 1200 volumes per run). The full imaging protocols can be found online at http://protocols.humanconnectome.org/HCP/3T/imaging-protocols.html.

Although the scanning parameters used to acquire the patient dataset differed from those used to acquire the HCP dataset, the concern of a methodological bias is mitigated by the fact that we did not compare groups directly; rather, we compared within-group longitudinal changes between the two groups [i.e., differences (groups) of differences (time points)]. As such, given that data at both time points for a given member of each group were acquired with the same parameters, one would only expect the presence of biases if one set of scanning parameters was inherently more sensitive towards detecting connectivity-based differences over time. In this case, our method, comprising fewer acquired volumes, was less sensitive, whereas the HCP method, comprising more acquired volumes, was more sensitive [23]. Thus, the scanning parameters applied to the patient sample were more statistically conservative and therefore less likely to yield false positives.

#### Neuroimaging data analysis

Analysis of the acquired neuroimaging data was carried out with the FMRIB Software Library (FSL, version 6.0) [24, 25], the Connectome Workbench (version 1.4.2) [26, 27] and the CoSMoMVPA toolbox [28] for MATLAB R2018b (The Mathworks, Natick, USA). Additionally, the HCP data had already been pre-processed using tools from FreeSurfer, FSL and the Connectome Workbench at the time we used it; see [22] for details on this procedure.

#### Pre-processing

Pre-processing steps included brain extraction [29], slice-time correction, and motion correction with respect to the middle volume of each run (using trilinear interpolation with 6 degrees of freedom (DOFs) [30]). We did not apply spatial smoothing, as its implementation has recently been shown to artificially increase the similarity of networks between subjects in functional connectivity analyses [31]. Additionally, we did not apply temporal filtering, which would have interfered with subsequent automatic removal of motion artifacts (see below).

Each patient’s functional scan was then linearly co-registered to the native high-resolution anatomical scan using a rigid body (6 DOFs) transformation. For statistical comparisons, we also performed a second, non-linear, co-registration of each functional scan to MNI152 2 mm standard space. Because non-linear registrations are only reasonable within the same modalities (e.g., structural, functional, etc.), we first implemented a 12 DOF affine transformation of the high-resolution anatomical scan to MNI152 2 mm space, which we then used as a starting point for the non-linear registration of the functional data to the standard space [32].

In the final pre-processing step, we corrected for head motion-induced artifacts using FSL’s Automated Removal Of Motion Artifacts (AROMA) tool, using its non-aggressive strategy [33]. This algorithm consists of three steps: first, independent component analysis (ICA) is applied to decompose the functional data into a set of spatially independent components. Second, those components that most likely relate to head motion are automatically identified based on four criteria (high-frequency content, maximum correlation with realignment parameters, edge fractions, and CSF fractions). Finally, the functional data is denoised by removing the variance explained by only the motion-related components. We performed this analysis on the functional data in native space, which we later transformed into MNI standard space using the previously generated transformation matrices.

The maps resulting from this procedure were in volumetric space, but a 2D representation of cortical data has several advantages over a 3D-representation, including enhanced alignment to the geometry of the cortex as well as increased statistical power [34]. Therefore, we transformed the preprocessed data into CIFTI files ﻿(Connectivity Informatics Technology Initiative, https://www.nitrc.org/projects/cifti/) using the Ciftify toolbox [35], a file format which renders cortical data on the surface while maintaining volumetric information of subcortical and cerebellar data. For simplicity, we will refer to the smallest units of our data as “voxels”, comprising both surface-based and cerebellar/subcortical representations.

As a control data set, we used minimally preprocessed data (both functional and structural scans) from the HCP-retest dataset, which are also provided in CIFTI format. This data has been preprocessed following the HCP functional preprocessing pipeline [22]. The main preprocessing steps were identical between our pipeline and the HCP pipeline, encompassing removal of spatial/distortion artifacts, surface generation, registration and alignment to standard space.

#### Assessing changes in brain network organization between relapse and remission

**Fig. 2:**
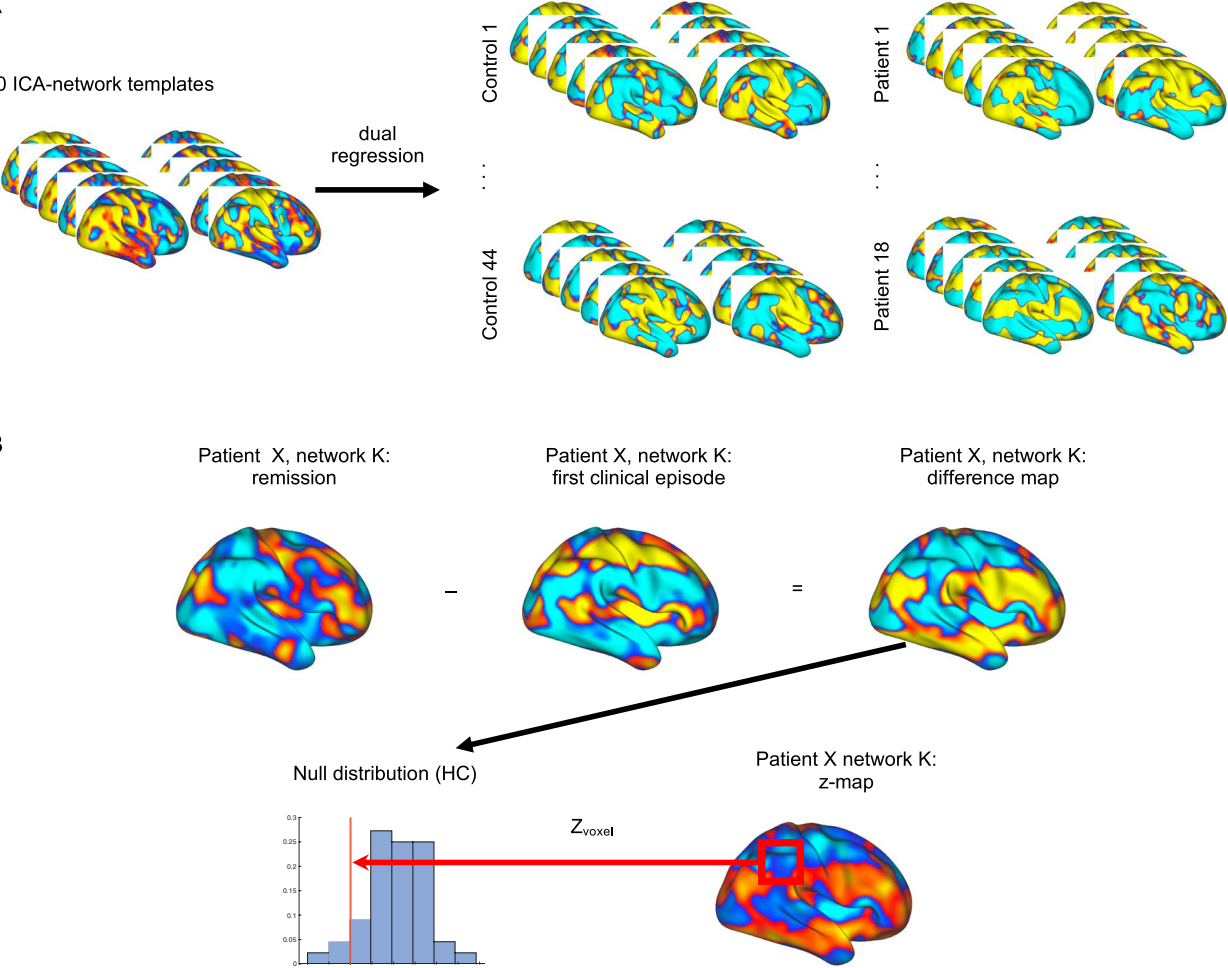
Calculating statistical maps of network changes. **A** First, we applied dual regression for a set of ten ICA-network templates to each control’s and each patient’s data sets, both for first clinical episode (baseline) and remission (follow-up). Next, (**B**, upper row) we computed the difference map for each patient between the two time points. Finally, (**B**, lower row) we converted the patients’ difference maps to z-maps, by calculating, for each voxel, the *z*-score with respect to the control group

**Fig. 3:**
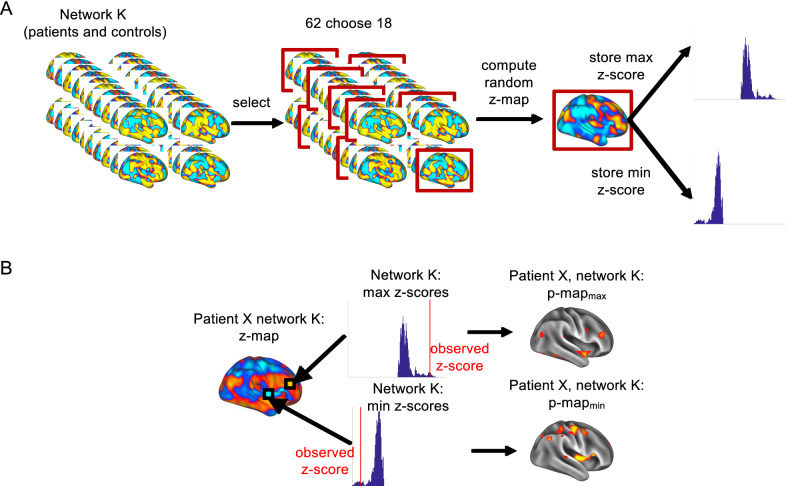
Statistical assessment. **A** Using a Monte-Carlo procedure, we generated null distributions to determine the expected degree of change in network organization between follow-up and baseline. **B** From these null distributions, we calculated empirical *p*-values for each voxel of the patients’ z-maps, which yielded regions reflecting a change in network organization

The central goal of our analysis (illustrated in Figs. 2 and 3) was to investigate whether the organization of patients’ brain networks changed between relapse and remission, compared to that of a sample of HC. Using dual regression [36] we generated subject-specific component maps based on ten previously published spatial components (from ICA, Table 2) and associated time courses [14] for both patients and HCs (Fig. 2A). Here, we used a version of this template in CIFTI file format [37]. Then, each of the ten component templates was regressed (as spatial regressors in a multiple regression) into each participant’s (i.e., each patient and each of the HCs) 4D space–time dataset. This procedure resulted in a set of ten subject-specific time series, one per spatial component. The resulting time series were used as temporal predictors in a multiple regression of each participant’s 4D dataset, resulting in ten subject-specific spatial maps of parameter estimates (PE). We then smoothed the PE maps with a Gaussian kernel of 5 mm FWHM (in order to improve the spatial correspondence between the data from patients and HCs). We applied this procedure to the scan during the first clinical episode (or baseline in HC) and the scan at remission (or follow-up in HCs). As we were interested in *changes* in brain networks, we computed the difference between the PE maps (for each of the ten templates separately) for each patient/HC from the two scans (i.e., we calculated remission minus first clinical episode for the patients and follow-up minus baseline for the HC, Fig. 2B, upper row). We then converted the patients’ difference maps, voxelwise, to *z*-score maps (Fig. 2B, lower row), using the mean and standard deviation from the difference maps (follow-up—baseline) of the 44 HCs. We computed these maps for each of the ten ICA-networks separately.

**Table 2:** Overview of the analyzed networks, according to Smith et al. [14]

Network name (abbreviation)	Network name (abbreviation), continued	Network name (abbreviation), continued
Medial visual areas (MVA)	Cerebellar Network (Crb)	Frontoparietal Network, right (rFP)
Occipital Pole (Occ)	Sensorimotor Network (SMN)	Frontoparietal Network, left (lFP)
Lateral Visual Areas (LVA)	Auditory Network (Aud)	
Default Mode Network (DMN)	Executive Control Network (ECN)	

In brackets we provide the abbreviations used throughout this text

In order to carry out statistical inference on the difference maps, we used a permutation testing scheme [38] based on the null hypothesis of no difference between patients and controls. The procedure was as follows (Fig. 3A): for each network, of all 62 analyzed subjects (combining 18 patients and 44 controls), we randomly selected the difference maps from 18 subjects (reflecting the number of patients in our sample) and calculated 18 *z*-scored difference maps, as described in the previous paragraph, from the distribution of the remaining 44 subjects (which could contain patients). From each of the resulting *z*-scored difference maps, we stored both the maximum and the minimum *z*-scores [to control for the inflated family-wise error rate (FWER)] in order to generate two separate null distributions for testing both increases and decreases of coherence. We repeated this process 10,000 times and subsequently compared the *z*-scored difference maps of the 18 patients (i.e. ten maps for each patient, reflecting the ten analyzed networks) to the permutation-based null distributions by counting how many values of the null distributions were more extreme than the observed *z*-scores in each voxel of the patients’ maps. Dividing this count by the number of permutations yielded FWER-corrected *p*-values (*p*_FWER_) (Fig. 3B). We considered voxels with a *p*_FWER_ lower than 0.025 (i.e., 0.05, two-tailed) to indicate changes in network organization that would not be predicted by chance. Hereinafter, we refer to networks in which we observed changes between the two time points as “altered networks”. For each altered network, if we identified one or more clusters of voxels with *p*_FWER_ < 0.025, we extracted those clusters using Workbench’s *cifti-find-clusters* algorithm. In the following sections, we refer to such clusters of voxels as regions of interest (ROIs).

#### Constructing adjacency matrices between altered networks and original networks

For all of the ROIs of a given altered network of a single patient, we identified to which of the ten *original* networks the ROIs belong, according to the ICA *z*-scores from the ten spatial components. This procedure yielded a 10 × 10 adjacency matrix of altered networks (rows) and original networks (columns). Thus, we counted the number of ROIs present for a given altered network and placed this count data in the corresponding column of the matrix. As we were interested in the number of patients that showed similar coherence changes, these data were binarized, so that, even if a patient had multiple ROIs reflecting coherence changes between the same two networks, this information appeared in the matrix as a one. This 10 × 10 binary adjacency matrix was populated for each patient and then summed across patients, yielding a group-level frequency matrix, which allowed us to perform group-level statistics to test our hypotheses. Such matrices were not necessarily symmetric because a pre- post change in an altered network that changed its connectivity with an original network does not imply that the original network also showed a pre- post change.

#### Assessing the one-with-one, one-with-many, and many-with-one hypotheses

Our hypotheses asked whether specific patterns appeared in the adjacency matrices, which would indicate systematicity between original networks and altered networks. Namely, we were interested in a one-with-one hypothesis (denoted by a single square in the adjacency matrix, Fig. 1B), a one-with-many hypothesis (denoted by a row in the adjacency matrix), and a many-with-one hypothesis (denoted by a column in the adjacency matrix). Thus, we needed to determine whether the frequencies for a single square or the pattern of frequencies for a given column or row were unlikely to occur by chance, which we carried out using a binomial test and Monte Carlo procedures.

To this end, we first calculated the expected number of patients that would contribute to a given cell of the matrix, which we obtained by summing all 100 frequencies of the group-level matrix (separately for coherence increases and decreases) and then dividing this sum by the number of elements of the matrix. This procedure yielded 1.99 patients for the increases and 2.58 patients for the decreases. Using these expected counts, we then ran a binomial test for each cell of the matrices to determine the probability of the observed number of patients, given the total number of patients that participated in the experiment. These *p*-values were then converted to *z*-scores (using a mean of 0 and standard deviation of 1) for ease of visually presenting heatmaps. The *z*-scores were ultimately thresholded at a value of 1.96 (i.e., *p* < 0.025) to be able to determine whether any column-, row, or cell-effects were present by counting how many elements in each column and row survived the threshold.

Assessing potential one-with-one effects was carried out by seeking lone cells with a *p*-value lower than the Bonferroni-corrected threshold (to account for the inflated family-wise error rate [FWER] from performing 100 tests in two matrices) of *p* < 0.025/200 (i.e., *z* > 3.6623). Assessing potential one-with-many or many-with-one effects (i.e., row- and column-effects, respectively) was carried out by first counting how many elements of a given row or column survived a threshold of *p* < 0.025 (i.e., *z* > 1.96) and then running a Monte Carlo procedure to determine how many elements of a row or column would be predicted by chance. This randomization procedure consisted of shuffling each patient’s binary matrix, recomputing the group-level frequency matrix, recomputing the binomial test, thresholding the resulting random *p*-values at *p* < 0.025, summing the number of elements per row and column, and storing the maximum row and column sums (also to control for the inflated FWER from ultimately testing for multiple column and row effects). We repeated this procedure 100,000 times yielding two distributions of maximum expected elements per column (once for coherence increases, once for coherence decreases) and two distributions of maximum expected elements per row (again, for coherence increases and decreases separately). We used these maximum sums to determine whether the observed number of elements comprising a column- or row-effect in the original data (following thresholding) were not likely to be predicted by chance.

#### Follow-up individual-level analysis

Given that our results pointed to unexpected commonalities across patients involving the Cerebellar Network and Executive Control Network, we opted to carry out a follow-up analysis to determine whether such effects were robustly present in all patients rather than merely being driven by a minority of the sample. To this end, as each ROI could belong to a different network (see above), for each patient we counted how often each “original network” appeared in the set of ROIs, ultimately yielding an individual-level probability distribution of the ten networks. These probabilities were gathered separately for coherence increases and decreases and then arcsine transformed before being submitted to a two-factor repeated-measures analysis of variance (ANOVA) with factors Coherence and Network. This procedure was repeated but instead counting how often each “altered network” appeared in order to also obtain individual-level probability distributions for the altered networks.

## Results

Our primary goal was to investigate whether there were regions in the brain that changed their coherence to functional brain networks (obtained via dual-regression ICA) between the first clinical episode (pre) and remission (post) in MS patients. We addressed this question by investigating ten networks (Table 2) for each patient, analyzing increases and decreases of functional coherence separately. To determine when pre-post changes in coherence differed from the expected degree of change present in a group of healthy controls, we employed Monte Carlo procedures, which revealed networks that differed between the first clinical episode and remission. We used these patterns of network alterations to test hypotheses regarding whether there exists systematicity across patients with respect to the networks that change and the networks to which they change.

### Clinical scores, but not lesion load, change between first clinical episode and remission

The median EDSS score of our patient sample was 2.25 (interquartile range = 1.0) at first clinical episode and 1.0 (interquartile range = 0.50) at remission. The Wilcoxon signed-rank test confirmed the decrease in EDSS scores over time (*z* = 53.50, *p* = 0.0059). On the other hand, we were unable to find differences in T2 lesion load (*t*_16_ = 0.4364, *p* = 0.6684) between the two time points.

### Many-with-one and one-with-many: coherence changes for the cerebellar and executive control networks

**Fig. 4:**
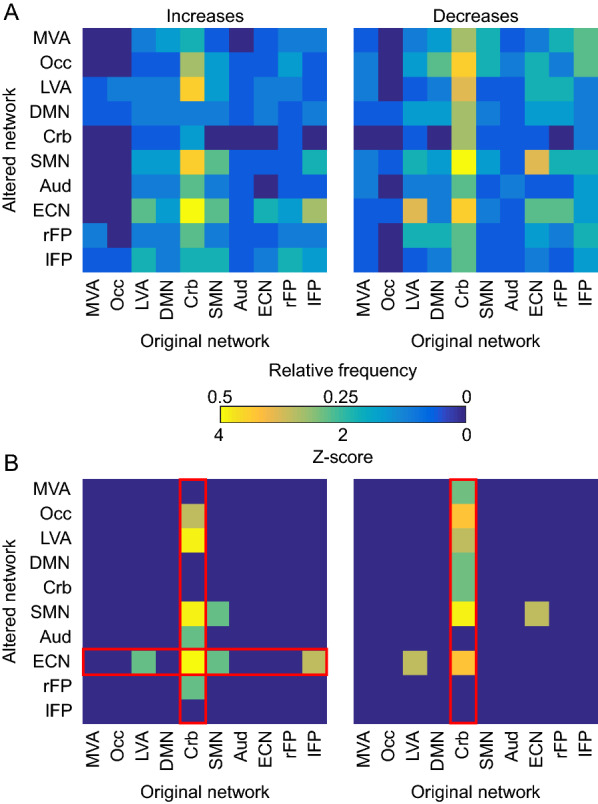
Main results. **A** Relative frequencies depicting how many patients had a particular altered network that changed its coherence with a particular original network. **B** Thresholded *z*-scores (at *z* > 1.96) from inverting *p*-values obtained via a binomial test. Following all statistical procedures, the results provide support for the many-with-one hypothesis, as several altered networks across patients appeared to both increase and decrease their coherence with the Cerebellar Network. There was additional evidence for the one-with-many hypothesis, in that coherence increased between the ECN and a set of other networks. Column and row effects that surpassed the respective FWER-corrected threshold of *p* < 0.05 (two-tailed) are highlighted by the red contours

The Monte Carlo procedures we used to test the one-with-many (row-effects) and many-with-one (column-effects) hypotheses revealed evidence for both hypotheses. Evidence for the many-with-one hypothesis manifested with respect to the Cerebellar Network (Crb) in terms of both increased (six of ten networks present, *p*_FWER_ = 9.9 × 10^−6^) and decreased coherence (seven of ten networks present, *p*_FWER_ = 9.9 × 10^−6^) (Fig. 4). For the coherence increases, the networks contributing to the column-effect were the Occipital Pole (Occ, *z* = 2.8597, *p* = 0.002), Lateral Visual Areas (LVA, *z* = 3.906, *p* = 4.69 × 10^−5^), Sensorimotor Network (SMN, *z* = 3.906, *p* = 4.69 × 10^−5^), Auditory Network (Aud, *z* = 2.3105, *p* = 0.01), Executive Control Network (ECN*, z* = 4.4116, *p* = 5.13 × 10^−6^), and right Frontoparietal Network (rFP, *z* = 2.3105, *p* = 0.01). For the coherence decreases, the networks contributing to the column-effect were Medial Visual Areas (MVA, *z* = 2.356, *p* = 0.009), Occ (*z* = 3.3809, *p* = 3.6 × 10^−4^), LVA (*z* = 2.8749, *p* = 0.002), Default Mode Network (DMN, *z* = 2.356, *p* = 0.009), Crb (*z* = 2.356, *p* = 0.009) (SMN (, *z* = 3.8778, *p* = 5.3 × 10^−5^), and ECN (, *z* = 3.3809, *p* = 3.6 × 10^−4^) (Fig. 4).

Evidence for the one-with-many hypothesis consisted of the ECN increasing its coherence with four of ten networks (*p*_FWER_ =  = 2.9 × 10^−4^): the LVA (*z* = 2.31, *p* = 0.01), Crb (*z* = 4.4116, *p* = 5.13 × 10^−6^), SMN (2.31, *p* = 0.01), and lFP (*z* = 2.8597, *p* = 0.002) (Fig. 4). The *z*-scores and *p*-values presented in this section for the individual networks came from the binomial test.

Moreover, the binomial test did not reveal evidence for the “one-with-other” hypothesis between any networks that were not already contributing to a “one-with-many” or “many-with-one” effect.

### Robust coherence changes of the cerebellar network in vast majority of patients

**Fig. 5:**
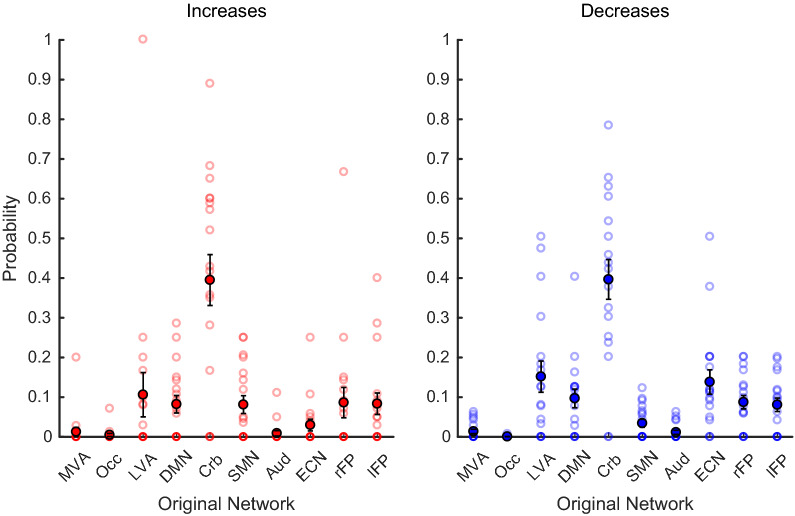
Group-averaged probabilities: original networks. The probability that each of the 18 patients’ ROIs, which showed coherence increases (red) and decreases (blue) with the altered network(s), belonged to a given one of the ten component networks. The Cerebellar Network was the most probable original network, appearing with a probability of ~ 39% for coherence increases (present in 14/18 patients) and ~ 39% for coherence decreases (present in 16/18 patients). For both panels, translucent circles reflect individual results, while opaque circles reflect the group -average. Error bars depict standard error of the mean

Our individual-level analysis revealed that the unexpected commonality of the Cerebellar Network altering its functional coherence with other networks was not driven by only a few patients. Rather, the Cerebellar Network was the most-likely (or equally-most-likely) “original network” [mean probability =  ~ 39 ± 6% (SEM)] for coherence increases (Fig. 5, left), occurring in 13 patients (i.e., ~ 72% of the sample; *p* = 4.31 × 10^−11^, from a binomial test *B*(18, 0.1056); Additional file 1: Figure S1). A fourteenth patient also showed coherence increases involving the Cerebellar Network, though it was not the most likely original network. With respect to the coherence decreases (Fig. 5, right), the Cerebellar Network was the most-likely original network (mean probability =  ~ 39 ± 5% [SEM]), occurring in 14 patients (i.e., ~ 78% of the sample; *p* = 5.82 × 10^−12^, from a binomial test *B*(18, 0.1167); Additional file 1: Figure S2). Two additional patients showed coherence decreases involving the Cerebellar Network, though it was not the most likely original network. The degree of coherence changes in the cerebellar network did not correlate with the changes in the EDSS scores (see Additional file 1). These observations were supported by the results from the ANOVA, which revealed differences between networks (*F*_9,153_ = 27.1, *p* = 1.49 × 10^–27^) and coherence directions (*F*_1,17_ = 6.15, *p* = 0.0239), but not an interaction between the two factors (*F*_9,153_ = 1.28, *p* = 0.2528). Moreover, post-hoc achieved power analyses using G*Power [39] revealed statistical power of 99.9985% for having found the effect of coherence increases involving the Cerebellar Network in 13 of 18 patients (needing only to be present in four patients, given an expected probability of 0.1056). Similarly, achieved statistical power was 99.9991% for having found the effect of coherence decreases involving the Cerebellar Network in 14 of 18 patients (needing only to be present in five patients, with an expected probability of 0.1167).

**Fig. 6:**
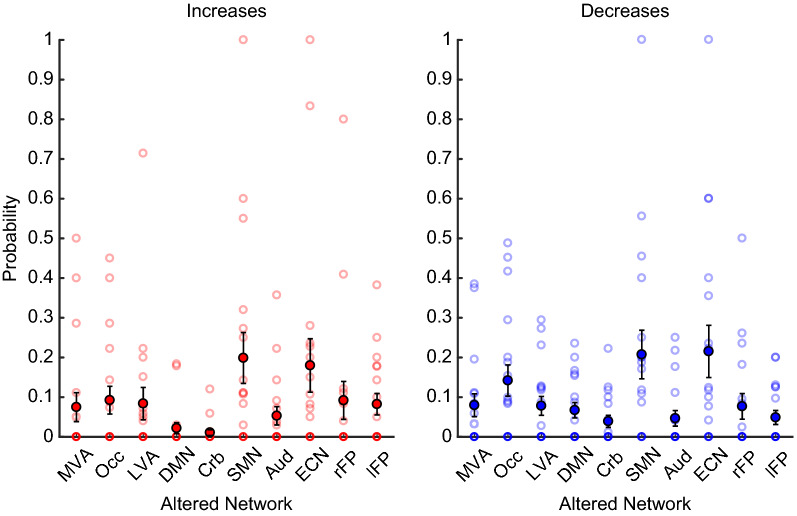
Group-averaged probabilities: altered networks. The probability that each of the 18 patients’ networks showed coherence increases (red) or decreases (blue), with respect to the sample of healthy controls. For both the coherence increases and decreases, the Sensorimotor and Executive Control Networks were most likely to be altered, appearing with a probability of ~ 20%. For both panels, translucent circles reflect individual results, while opaque circles reflect the group -average. Error bars depict standard error of the mean

With respect to the individual-level probabilities of the original networks, the ANOVA revealed differences between networks (*F*_9,153_ = 3.13, *p* = 0.0017) and between coherence directions (*F*_1,17_ = 5.04, *p* = 0.038) but not an interaction between the two factors (*F*_9,153_ = 0.84, *p* = 0.58) (Fig. 6, Additional file 1: Figures 3, 4).

## Discussion

### “One-with-many effect”: executive control network

Our analysis revealed evidence for the “one-with-many” hypothesis, in that the functional coherence of the ECN increased with respect to the LVA, Crb, SMN, and lFP. This finding stands in partial contrast to previous work, which has shown, for example, that decreases in functional connectivity between the ECN and the DMN at one time point (as compared to controls) predict increased clinical disability (i.e., higher EDSS scores) [40]. Our findings suggest instead that a relative increase of the ECN with other networks *is* compatible with remission (i.e., lower EDSS scores). One important distinction, however, is that our sample consisted of patients suffering from their initial clinical episode who were scanned during that first episode and again in remission, thereby allowing us to measure changes *over time* within patients. In contrast, such prior work investigated relapsing–remitting (RRMS) patients that were scanned only once; as such, the authors were only able to describe differences with respect to healthy controls but not which changes may have occurred within a given patient. It is therefore possible that the connectivity behavior of the ECN differs at different stages of MS.

### “Many-with-one-effect”: cerebellar network

Most critically, we found evidence for the “many-with-one” hypothesis, in that functional coherence changed (both increasing and decreasing) between a set of networks and the Cerebellar Network (Fig. 4), and that this result is rather robust at the individual level (i.e., present in more than 70% of patients), despite the clinical heterogeneity of the sample. To demonstrate the robustness of this effect, we carried out a split-half analysis in which we compared the results from the first nine patients with the results from the second nine patients (see Additional file 1: Fig. S1).

Such a finding raises the question of whether there is some capacity of the Cerebellar Network that allows RRMS patients to overcome their symptoms; this idea is supported by converging evidence that has tied functional and structural aspects of the cerebellum to the severity of MS disease progression. For example, MS patients with early cerebellar dysfunction tend to develop disability more quickly [41]. Similarly, Kutzelnigg and colleagues showed that the extent of cerebellar cortical demyelination is associated with overall MS pathology [42]. Recent cross-sectional studies investigating functional connectivity of MS patients demonstrated a relationship between symptom severity and alterations of the cerebellum’s functional connectivity [43–45]. Our findings extend such studies by providing additional evidence of functional changes with respect to the Cerebellar Network when measured at relapse and remission, and, more importantly, yield a new perspective that functional connectivity is altered between the Cerebellar Network and a variety of other networks, irrespective of clinical symptoms. These findings give rise to the idea that the Cerebellar Network may play a guiding role in recovery from diverse MS symptoms in the early disease course and may protect against permanent disability.

Despite these findings, the question of how the Cerebellar Network may alleviate MS symptoms remains open. One mechanism by which the Cerebellar Network might be involved in remission is that of learning. Building off of the original theory of cerebellar learning [46], the current Albus-Marr-Ito theory of cerebellar learning posits that information from sensory consequences of actions is directly conveyed to the cerebellum via the inferior olive [47, 48]. Additionally, copies of any efferent motor program are propagated to the cerebellum via collaterals from cortical neurons [49, 50]. Thus, as the cerebellum can access both efferent plans and afferent consequences, it has the potential to register when there is a deviation between the two and correct if necessary [51]. Though Marr originally thought that the cerebellum was involved only in learning motor programs, recent work has shown that the cerebellum receives input from distributed cortical areas and is involved in a broad range of cognitive functions beyond movement [52].

With respect to MS and the idea of a protective role of the cerebellum, earlier work provides evidence that such motor and/or cognitive learning may underlie the improvement of symptoms in MS patients. For example, using fMRI Saini and colleagues observed activation patterns in clinically stable MS patients that are reportedly found in healthy controls during motor learning [53]. Similarly, MS patients, whose performance on cognitive tasks successfully improved following cognitive training programs (as compared to MS patients that did not receive cognitive training), showed increased activation within the cerebellum [54] and altered functional connectivity of the cerebellum [55]. Thus, a link between learning, altered cerebellar recruitment, and MS symptom decline may explain the effectiveness of cognitive/motor training programs in MS rehabilitation [56, 57].

## Limitations and future outlook

One limitation of our study concerns the interpretation of the “many-with-one” effect that we observed implicating the Cerebellar Network in processes underlying remission in MS. By definition, a “many-with-one” effect is composed of multiple “one-with-one” effects that surpassed a particular threshold and, together, form the pattern of a column. Because these columnar patterns were statistically unlikely to occur by chance, we interpreted them as evidence for the “many-with-one” hypothesis, rather than a set of isolated effects, each favoring the “one-with-one” hypothesis. Regardless, we cannot rule out the possibility that treating these effects in isolation would be the correct interpretation. For example, consider a scenario in which different patient subtypes have different altered networks that change their coherence to the Cerebellar Network. By analyzing these patients as a single group, one would observe what looks like a “many-with-one” effect, which is merely the result of the heterogeneous sample. However, our sample size is too small to differentiate subtypes. As such, this idea should be scrutinized in larger studies that can test the effectiveness of methods such as ours in identifying patient subtypes, thereby potentially improving personalized treatment strategies [58].

Despite this interpretational shortcoming of the “many-with-one” effect, there remain the facts (1) that functional coherence was commonly altered between a variety of networks and the Cerebellar Network and (2) that the Cerebellar Network was either the most likely original network or tied for most likely original network (in at least 70% of the patients for both the coherence increases and decreases). Therefore, this unexpected commonality across patients still speaks in favor of our general interpretation, providing evidence that the Cerebellar Network’s specific role in MS should be further investigated, for example, as a possible target for symptom-alleviating therapies.

Additionally, the open question regarding the *meaning* of increases and decreases of functional coherence of the Cerebellar Network remains unknown. Such differences in functional connectivity are believed to reflect different underlying mechanisms [59]; with respect to our findings, it is possible that the coherence increases and decreases reflect different types of plasticity at the synaptic level, specifically long-term potentiation (LTP) and long-term depression (LTD). Both of these learning mechanisms have been observed for cerebellar neurons [60–63], and LTP has been associated with symptom decline in MS patients [64, 65]. Moreover, prior work has shown that LTP increases functional connectivity in the rat cortex [66, 67]; it is thus conceivable that LTD analogously decreases functional connectivity, but this relationship has yet to be experimentally demonstrated. Consequently, alterations of cerebellar functional connectivity in MS patients may underlie neuroplastic changes that could theoretically predict symptom severity.

## Conclusion

In summary, we investigated changes of functional connectivity in MS patients between their first clinical episode and remission using ICA-based rs-fMRI. Our analyses revealed that, the Cerebellar Network and a heterogeneous set of other networks across different MS patient tended to alter their functional coherence to one another, and that there is approximately a 39% chance of observing functional coherence changes in the Cerebellar Network for a given patient following the initial clinical episode. This finding suggests that the Cerebellar Network may be functionally compensating for structural damage to distributed brain regions, which could help explain the oft-observed mismatch between structural damage and clinical disability [68]. Furthermore, these alterations in functional coherence manifested as both increases and decreases, which may reflect neuroplastic mechanisms of learning. Within this framework, subsequent studies could investigate the cerebellum’s capacity for such learning mechanisms as a potential biomarker that predicts an individual’s clinical progression; more generally, future work could seek to understand the specific role of the cerebellum in the presence of distributed damage to the structural and functional architecture of the brain, especially at different stages of MS.

## Supplementary Information

**Additional file 1:** **Figure S1.** Probability that a given network is the “original network”, whose functional coherence is changed in MS patients following their entrance into remission. Open circles are data from individual patients; closed circles depict the mean, and error bars are SEM. **(A)** Results from the first set of 9 patients. **(B)** Results from the second set of 9 patients. Note that in both cases the cerebellar network is, by far, the most likely network to change its functional coherence.

## Data Availability

Statistical data generated during the analyses presented in this study will be made available to readers upon reasonable request to the corresponding author, but raw/preprocessed data acquired from patients in this study cannot be shared due to the European Union’s General Data Protection Regulation.

## Abbreviations

Aud: Auditory Network
ANOVA: Analysis of variance
AROMA: Automated Removal of Motion Artifacts
BW: Bandwidth
CIFTI: Connectivity Informatics Technology Initiative
CSF: Cerebrospinal fluid
Crb: Cerebellar Network
DMN: Default Mode Network
DOF: Degree of freedom
ECN: Executive Control Network
EDSS: Expanded Disability Status Scale
EPI: Echo-planar imaging
FA: Flip angle
FC: Functional connectivity
FLAIR: Fluid-attenuated inversion recovery
FOV: Field of view
FWER: Family-wise error rate
HC: Healthy control(s)
HCP: Human Connectome Project
ICA: Independent component analysis
lFP: Frontoparietal Network, left
LVA: Lateral Visual Areas
LTD: Long-term depression
LTP: Long-term potentiation
MVA: Medial Visual Areas
MNI: Montreal Neurological Space
MPRAGE: Magnetization-prepared rapid acquisition gradient echo
MS: Multiple sclerosis
Occ: Occipital Pole
PE: Parameter estimates
rFP: Frontoparietal Network, right
ROI: Region of interest
rs-fMRI: Resting-state functional MRI
SEM: Standard error of the mean
SMN: Sensorimotor Network
TE: Echo time
TR: Time to repeat
VR: Voxel resolution
