# Supplementary material for: Early remission in multiple sclerosis is linked to altered coherence of the Cerebellar Network
Source: J Transl Med. 2022 Oct 27;20:488. doi: 10.1186/s12967-022-03576-4 (PMC9615296; doi:10.1186/s12967-022-03576-4)
Supplement: Supplementary file 1 — Additional file 1: Figure S1. Probability that a given network is the “original network”, whose functional coherence is changed in MS patients following their entrance into remission. Open circles are data from individual patients; closed circles depict the mean, and error bars are SEM. (A) Results from the first set of 9 patients. (B) Results from the second set of 9 patients. Note that in both cases the cerebellar network is, by far, the most likely network to change its functional coherence. [file 12967_2022_3576_MOESM1_ESM.docx]

Additional file

| A) change of coherence between first clinical episode and remission in patients 1-9   |
| --- |
| B) change of coherence between first clinical episode and remission in patients 10-18   |

Figure S1**.** Probability that a given network is the “original network”, whose functional coherence is changed in MS patients following their entrance into remission. Open circles are data from individual patients; closed circles depict the mean, and error bars are SEM. **(A)** Results from the first set of 9 patients. **(B)** Results from the second set of 9 patients. Note that in both cases the cerebellar network is, by far, the most likely network to change its functional coherence.

Supplementary Analysis

To test an association between changes of EDSS and extent of cerebellar network changes, we ran the following analysis: For a given patient, we averaged together all the delta-FC values from ROIs that corresponded to the cerebellar network as the "original" network. This resulted in a vector of delta-FC values for the coherence increases and the coherence decreases (per patient), which we correlated with the delta-EDSS scores. The results are as follows:

r = -0.17, p = 0.59 for the coherence increases

r = 0.159, p = 0.62 for the coherence decreases

We did not find evidence of a relationship between the EDSS scores and the actual coherence changes in the cerebellar network at this point. This null result is not surprising because of the few patients with EDSS scores from both sessions. The relationship between EDSS scores and coherence changes needs further evaluation in larger studies.
